# Genome-wide association analysis and genomic prediction of salt tolerance trait in soybean germplasm

**DOI:** 10.3389/fpls.2024.1494551

**Published:** 2024-11-18

**Authors:** Rongqing Xu, Qing Yang, Zhi Liu, Xiaolei Shi, Xintong Wu, Yuehan Chen, Xinyu Du, Qiqi Gao, Di He, Ainong Shi, Peijun Tao, Long Yan

**Affiliations:** ^1^ Hebei Laboratory of Crop Genetics and Breeding, National Soybean Improvement Center Shijiazhuang Sub-Center, Huang-Huai-Hai Key Laboratory of Biology and Genetic Improvement of Soybean, Ministry of Agriculture and Rural Affairs, Institute of Cereal and Oil Crops, Hebei Academy of Agricultural and Forestry Sciences, Shijiazhuang, China; ^2^ College of Agronomy, Hebei Agricultural University, Baoding, China; ^3^ Department of Horticulture, University of Arkansas, Fayetteville, AR, United States

**Keywords:** soybean, salt stress, genome-wide association study, genomic prediction, germplasm

## Abstract

**Introduction:**

Soybean is an important protein and oil crop, and improving yield has traditionally been a major breeding goal. However, salt stress is an important abiotic factor that can severely impair soybean yield by disrupting metabolic processes, inhibiting photosynthesis, and hindering plant growth, ultimately leading to a decrease in productivity.

**Methods:**

This study utilized phenotypic and genotypic data from 563 soybean germplasms sourced from over 20 countries. Employing four distinct models—we performed a genome-wide association study (GWAS) using four models, including MLM, MLMM, FarmCPU, and BLINK in GAPIT 3, we conducted a Genome-Wide Association Study (GWAS) to identify single nucleotide polymorphism (SNP) associated with salt tolerance in soybean. Subsequently, these identified SNP were further analyzed for candidate gene discovery. Using 34,181 SNPs for genomic prediction (GP) to assess prediction accuracy.

**Results:**

Our study identified 10 SNPs significantly associated with salt tolerance, located on chromosomes 1, 2, 3, 7, and 16. And we identified 11 genes within a 5 kb window upstream and downstream of the QTLs on chromosomes 1, 3, and 16. Utilizing the GWAS-derived SNP marker sets for genomic prediction (GP) yielded r-values greater than 0.35, indicating a higher level of accuracy. This suggests that genomic selection for salt tolerance is feasible.

**Discussion:**

The 10 identified SNP markers and candidate genes in this study provide a valuable reference for screening and developing salt-tolerant soybean germplasm resources.

## Introduction

Soybean is widely cultivated in the world, it is an important food and economic crop, ranking sixth in global food crop production (Du et al., 2023; Lu et al., 2020; Wang et al., 2023). As the most significant legumes globally, soybean is rich in protein, oil, isoflavones, and dietary fiber, providing high nutritional value (Graham and Vance, 2003). It also offer health benefits, including enhanced human immunity, prevention of cardiovascular diseases, and potential anti-aging effects. With improving living standards, the demand for soybean products has steadily increased. However, the average global yield of soybeans, approximately 2.5 to 3 tons per hectare, is insufficient to meet this growing demand. As a result, increasing soybean yield has become a priority for breeders.

Soil salinity, a major abiotic stress factor, significantly inhibits seed germination, growth, and nodule formation in soybeans (Ondrasek et al., 2011; Singleton and Bohlool, 1984). Data from the Food and Agriculture Organization of the United Nations and the United Nations Environment Program reveal that over 950,000 square kilometers of land worldwide have been degraded to saline-alkali conditions, accounting for more than 8% of the global land area (Beecher, 1994). Therefore, breeding salt-tolerant soybean varieties is essential for enhancing soybean production.

There has been extensive research on the genes and quantitative trait loci (QTLs) associated with salt tolerance in soybeans. To date, 1,536 QTLs related to salt tolerance have been identified, primarily distributed across chromosomes 2, 3, 6, 8, 9, 12, 13, 14, and 17. Utilizing 196 soybean landraces and 184 families, Kan et al. (2016) identified 22 SSR markers tightly linked to salt tolerance during germination, as well as 11 QTL loci on chromosomes 2, 7, 8, 10, 17, and 18. Similarly, Chen et al. (2008) conducted QTL mapping for salt tolerance at the seedling stage in soybeans using a RIL population of 184 lines, identified eight QTL loci on chromosomes 2, 3, 7, 9, 11, 14, and 18. In another separate study, Huang (2013) conducted visual leaf scorch scoring under salt stress and identified 62 SNP markers on chromosomes 2, 3, 5, 6, 8, and 18 that were significantly associated with salt tolerance.

Genome-Wide Association Study (GWAS) has become a favored method for studying the association between complex traits and genetic variations across the genome due to its high efficiency and shorter time required for constructing populations. Kan et al. (2016) conducted a GWAS of four salt tolerance indices using 191 soybean germplasms genotyped with 1,142 SNPs and identified eight SNP markers and five candidate genes associated with salt stress. Zhang et al. (2019) performed salt inhibition seed germination experiments on 211 cultivated soybean germplasms and conducted a GWAS of four salt tolerance indices with 207,608 SNPs from the NJAU 355 K SoySNP database (CMLM model). They detected 92 trait markers on chromosomes 1, 8, 11, 13, 14, 15, 16, 18, and 19. Further integration of QTL mapping results from 184 RILs and gene expression analysis identified a candidate gene, *Glyma.08g102000*, which belongs to the cation diffusion facilitator (CDF) family, for salt tolerance. Transgenic verification confirmed the gene’s role in regulating salt tolerance. In another study, Patil et al. (2016) measured chloride concentration and chlorophyll content in the leaves of 106 soybean lines at the V2 stage and conducted association analysis with 37,000 SNP markers from the SoySNP50K database. They identified 30 SNPs on chromosome 3 significantly associated with chlorophyll content and leaf wilting degree. Zeng et al. (2017) used 283 soybeangermplasm collected worldwide and measured chloride concentration and chlorophyll content in leaves at the V1 stage as salt tolerance indicators. They conducted a GWAS using 33,000 SNP markers from the SoySNP50K dataset (Song et al., 2015) and identified 45 SNPs on chromosomes 2, 3, 7, 8, 10, 13, 14, 16 and 20, and 31 SNPs on chromosome 3 significantly associated with salt tolerance.

Genomic Prediction (GP) can significantly accelerate breeding process. Molecular breeding of crops, particularly through Genomic Selection (GS), is an approach in crop genetic improvement. Compared to marker-assisted selection (MAS), GS can capture genetic effect, shorten breeding cycle and improve breeding efficiency (Hickey et al., 2017). Duhnen et al. (2017) reported an average prediction accuracy of 0.39 for soybean yield, and as high as 0.80 in some population (Jarquín et al., 2014). GP plays a crucial role in GS, allowing researchers to predict crop traits across various environments (Keller et al., 2020; Shikha et al., 2017).

Currently, there are 563 soybean germplasms with salt phenotypic data available in the United States Department of Agriculture (USDA) Germplasm Resources Information Network (GRIN) database (https://npgsweb.ars-grin.gov/gringlobal/descriptordetail?id=51054). Of these, 563 germplasms also have SNP genotypic data in SoyBase (https://www.soybase.org/snps/; Song et al., 2015). The purpose of this study is to utilize these phenotypic and genotypic data to conduct a GWAS to identify SNP markers associated with salt tolerance in soybean. Additionally, GP was performed to evaluate its potential application in selecting salt-tolerant lines for soybean breeding programs.

## Materials and methods

### Plant material

The 563 soybean germplasms from the USDA Germplasm Collection were used for this study. These germplasms were originally collected from 26 countries, including Japan (159 germplasms), China (86), India (59), South Korea (46), North Korea (28), South Africa (26), Nepal (20), Indonesia (16), United States (16), Suriname (13), Thailand (10), and 15 other countries (39), plus 45 germplasms of unknown origin (**Supplementary Tables 1A, B**).

### Phenotyping

The phenotypic data for salt tolerance reactions in 563 soybean germplasms were downloaded from the USDA GRIN website: https://npgsweb.ars-grin.gov/gringlobal/descriptordetail?id=51054. The experiments were conducted in Illinois, United States by Randy Nelson at the USDA Soybean Collection in Urbana, IL. The salt reaction was scored as tolerant (T) and susceptible (S) for each accession. ‘1’ indicating tolerance and ‘9’ indicating susceptibility were used to perform GWAS to identify SNP markers associated with salt tolerance in this study (**Supplementary Table 1A**).

### Genotyping

The germplasms were genotyped using Soy50K SNP Infinium Chips (Song et al., 2013). A total of 42,292 SNPs across 563 soybean germplasms were downloaded from SoyBase at https://soybase.org/snps/download.php (Song et al., 2015). For GWAS, 34,181 SNPs were selected after excluding those with more than 5% missing data, heterozygosity greater than 5%, and a minor allele frequency (MAF) less than 5%. These SNPs were distributed across all 20 chromosomes of the soybean genome (**Supplementary Figure 1**).

### Principal component analysis and genetic diversity

In this study, 34,181 SNPs were included in the principal component analysis (PCA) and genetic diversity analysis. PCA and genetic diversity were analyzed using GAPIT 3 (Wang and Zhang, 2021), with PCA components set from 2 to 10 and NJ tree settings from 2 to 10. Phylogenetic trees were drawn using the neighbor-joining (NJ) method in GAPIT 3. Genetic diversity was assessed for all 563 tested germplasms and their salt tolerance using (1) 34,181 SNPs in GAPIT 3, and (2) 10,000 randomly selected SNPs in MEGA 7 (Kumar et al., 2016). The phylogenetic trees were drawn using MEGA 7 based on the Maximum Likelihood method with the parameters described in Shi et al. (2016, 2017).

### Association analysis

GWAS was conducted using various models, including Bayesian-information and Linkage-disequilibrium Iteratively Nested Keyway (BLINK), Fixed and Random Model Circulating Probability Unification (FarmCPU), Generalized Linear Model (GLM), and Mixed Linear Model (MLM) in GAPIT 3 (Wang and Zhang, 2021). The analysis was performed on a panel of 563 germplasms using 34,181 SNPs. Multiple GAPIT models were utilized to identify robust and consistent SNP markers associated with salt tolerance in soybean. The significance threshold for germplasms was determined using Bonferroni correction of *P*-values with an α = 0.05 (0.05/SNP number). An LOD (logarithm of odds) value of 5.83 [Here, we use LOD instead of –log(*P*-value)] was used as the significance threshold based on the 34,181 SNPs.

### Candidate gene prediction

Candidate genes associated with salt tolerance were sought within a 5 kb vicinity on both sides of the significant SNPs, following the methodology outlined by Zhang HY, et al., 2016. The candidate genes were extracted from the reference annotation of the soybean genome assembly, Wm82.a2.v1, available at https://phytozome-next.jgi.doe.gov/info/Gmax_Wm82_a2_v1.

### Genomic prediction for genomic selection of salt tolerance

In this investigation, ridge regression best linear unbiased prediction (RR-BLUP) from the rrBLUP package (Endelman, 2011) and Bayesian models, including Bayes A (BA), Bayes B (BB), Bayes LASSO (BL), and Bayes ridge regression (BRR), implemented in the BGLR package, were employed for predicting genomic estimated breeding values (GEBV) in GP. The analysis was carried out using R software version 4.3.1 (https://www.r-project.org/). Additionally, GEBV prediction was conducted using genomic best linear unbiased prediction (gBLUP), composite BLUP (cBLUP), marker-assisted BLUP (maBLUP), and settlement of MLM under progressively exclusive relationship (SUPER) BLUP (sBLUP) methods, implemented in the GAPIT package. The effectiveness of genomic prediction using these approaches has been documented in prior research studies (Shi et al., 2021; 2022; Jarquín et al., 2014; 2017; Zhang JP, et al., 2016).

Genomic prediction (r-value) for salt tolerance was conducted across various soybean panels and scenarios. Firstly, GP was estimated using a training set to predict salt tolerance in the panel of 563 soybean germplasms. Predictions were estimated using four models: maBLUP, gBLUP, cBLUP, and sBLUP, utilizing all 34,181 SNPs in GAPIT3. Secondly, GP was executed using ten SNP sets: eight randomly selected SNP sets ranging from 10 to 10,000 SNPs, plus a GWAS-derived SNP marker set containing 10 markers (m10). These predictions were estimated using five GP models: BA, BB, BL, BRR, and rrBLUP. The prediction accuracy for salt tolerance was assessed using the average Pearson’s correlation coefficient (r) between the GEBVs and observed values in the validation set. Training and validation sets were randomly created 100 times, and the r-value was estimated for each iteration. The average r-value across the 100 iterations was then calculated for salt tolerance. In the GP scenarios, a higher r-value indicates greater prediction accuracy and better selection efficiency in GS, reflecting the reliability of the GP for salt tolerance.

## Results

### Evaluation of salt tolerance

**Figure 1:**
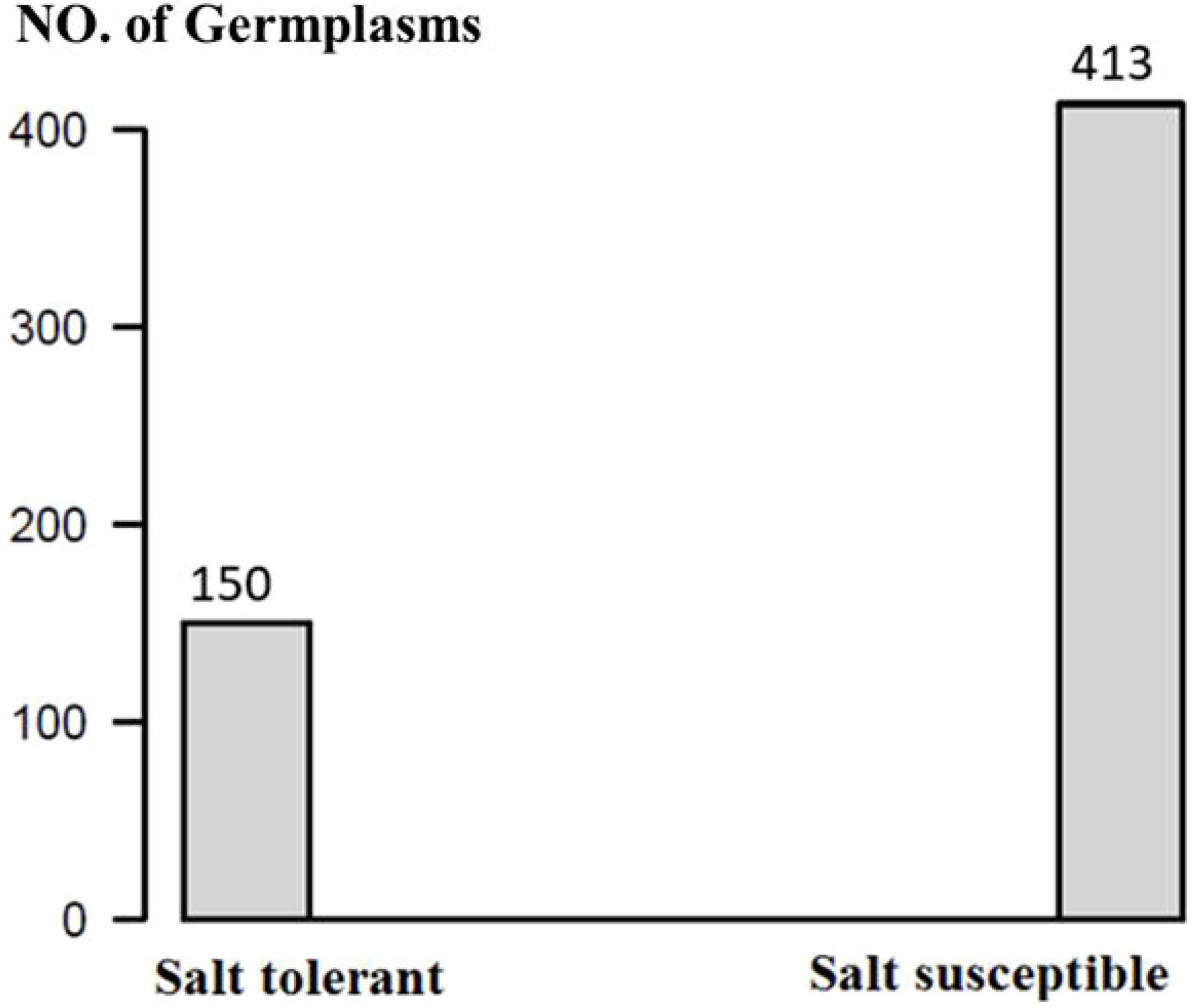
The distribution of salt tolerance reaction in 563 soybean germplasms.

The 563 soybean germplasms were divided into two groups, where 150 germplasms were salt tolerant and 413 were susceptible (**Figure 1**; **Supplementary Tables 1A, C**). These 150 salt-tolerant germplasms can be used as parents in soybean breeding programs to develop salt-tolerant lines.

### Genome-wide association study

**Figure 2:**
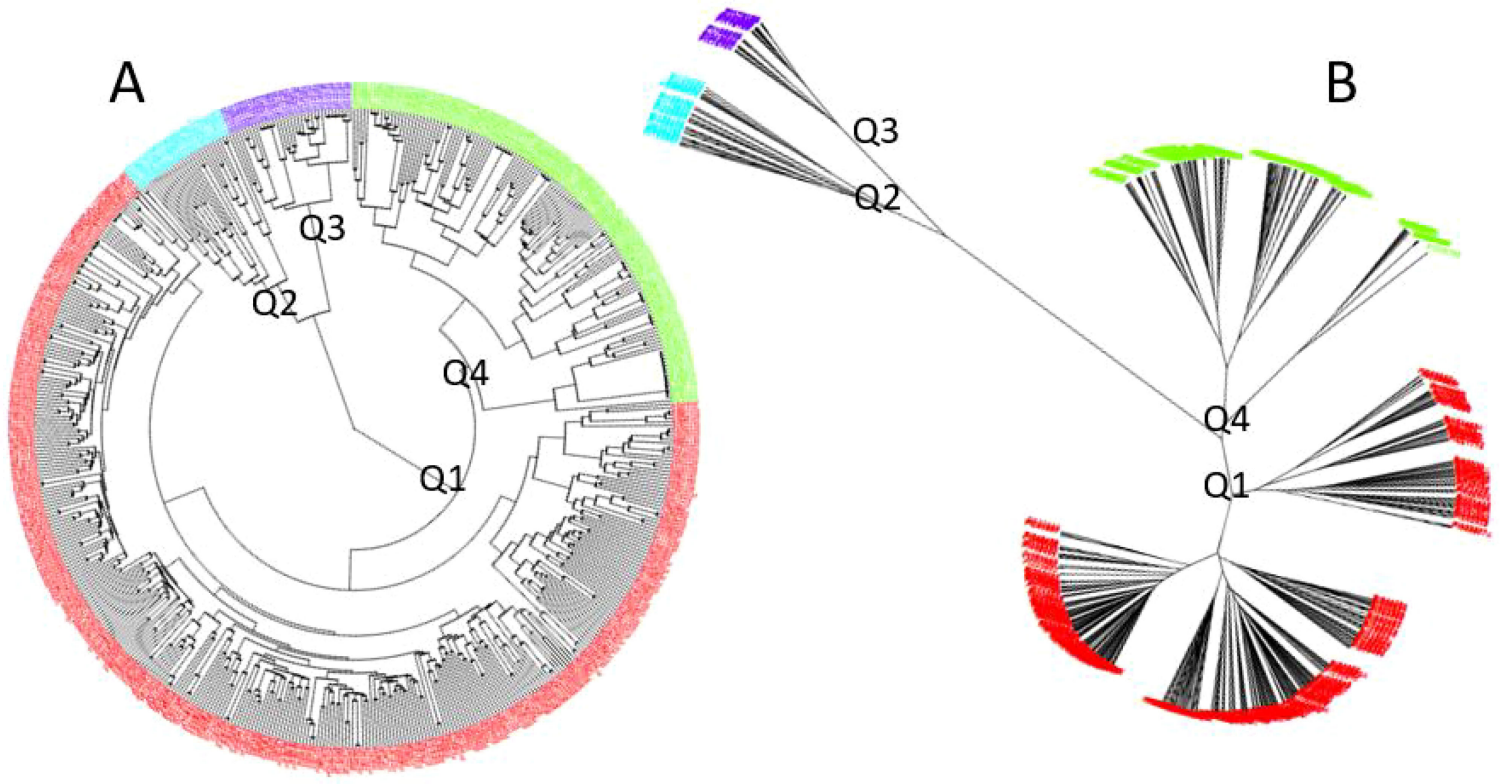
Population genetic diversity analysis in the association panel consisting of 563 USDA soybean germplasms: phylogenetic trees [**(A)**. fan and **(B)**. unrooted] drawn using the neighbor-joining (NJ) method in four sub-populations (Q1-Q4) by GAPIT3.

Using GAPIT 3, the 563 soybean germplasms were divided into four distinct clusters (subpopulations), labeled Q1 to Q4, based on the analysis of 34,181 SNPs (**Figure 2**; **Supplementary Figure 2**). The clustering was derived from the following analyses: (1) a 3D graphical plot of the principal component analysis (PCA) (**Supplementary Figure 2**, left), (2) a PCA eigenvalue plot (**Supplementary Figure 2**, right), and (3) phylogenetic trees constructed using the neighbor-joining (NJ) method (**Figure 2A**, ring – left and **Figure 2B**, no-root - right). Additionally, the kinship plot confirmed the existence of these four groups among the 563 germplasms (**Supplementary Figure 3**). Each was assigned to one of the four clusters (Q1 to Q4) (**Supplementary Table 1A**), and the resulting Q-matrix with four clusters was subsequently applied to the GWAS analysis.

**Figure 3:**
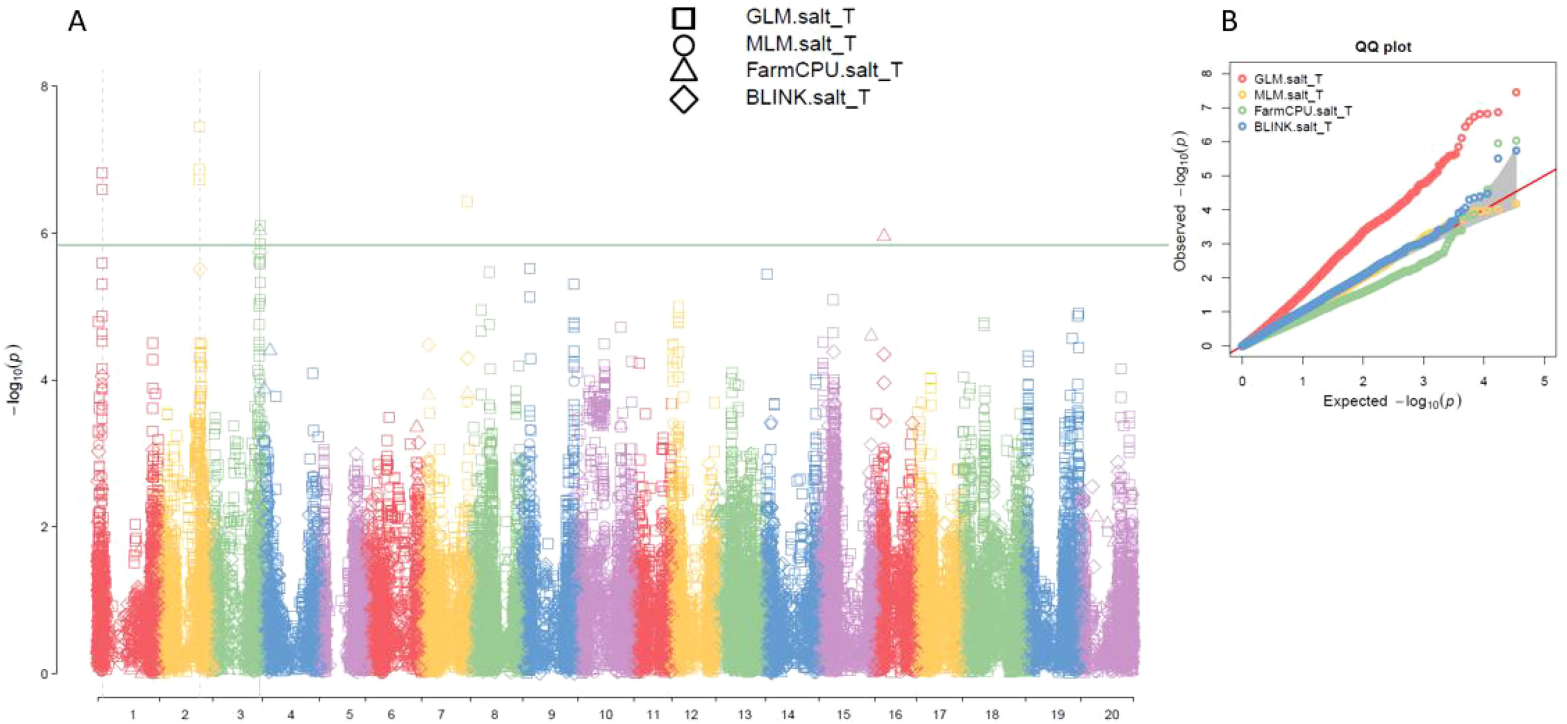
Multiple Manhattan plot **(A)** and QQ plot **(B)** of SNP significant level for salt tolerance among GLM, MLM, FarmCPU, and BLINK models in GAPIT3 in an association panel consisting of 563 germplasms. The Manhattan plot (left) illustrates soybean 20 chromosomes on the x-axis and LOD (-log(P-value)) values on the y-axis. The QQ plot (right) displays LOD (-log(P-value)) values on the x-axis and expected LOD (-log(P-value)) values on the y-axis.

Based on the analysis using four models (GLM, MLM, FarmCPU, and BLINK) in GAPIT 3, the multiple QQ plots showed a significant deviation from the expected distribution (**Figure 3**, right half; **Supplementary Figure 4**, right), indicating the presence of SNPs associated with salt tolerance. The multiple Manhattan plots, covering all 34,181 tested SNPs, revealed several SNPs with LOD values greater than 5.83, primarily located on chromosomes 1, 2, 3, 7, and 16, suggesting the SNPs were associated with salt tolerance in the panel (**Figure 3**, left half; **Supplementary Figure 4**, left).

Ten SNPs with LOD values greater than 5.83 were detected by at least one model (GLM or FarmCPU) in GAPIT 3 for salt tolerance (**Table 1**). Three SNP markers associated with salt tolerance, Gm01_4306329_ss715579436, Gm01_4312808_ss715579441, and Gm01_4336306_ss715579451, are in a region from 4,306,329 bp to 4,336,306 bp, with an interval of 30 kb on chromosome 1. The LODs of these SNPs were greater than 6.5 in the GLM model (**Table 1**). The other three SNP markers associated with salt tolerance, Gm02_36878905_ss715582154, Gm02_36940321_ss715582156, and Gm02_36991983_ss715582157, are located in a region from 36,878,905 bp to 36,991,983 bp on chromosome 2. These SNPs had an LOD greater than 6.7 in the GLM model and greater than 4.1 in a *t*-test (**Table 1**), indicating the presence of a QTL in this region. The two SNP markers, Gm03_43213208_ss715586397 and Gm03_43220331_ss715586399, are located at 43,213,208 bp and 43,220,331 bp, respectively, on chromosome 3. These SNPs showed an LOD greater than 5.83 in either the GLM or FarmCPU model and greater than 8.9 in t-tests (**Table 2**), suggesting a QTL in this region of chromosome 3 for salt tolerance. The SNP marker Gm07_41776639_ss715598058, located at 41,776,639 bp on chromosome 7, is associated with salt tolerance with an LOD of 6.44 in the GLM model and 5.94 in the t-test (**Table 2**). The SNP marker Gm16_7722217_ss715625494, located at 7,722,217 bp on chromosome 16, is associated with salt tolerance with an LOD of 5.95 in the GLM model and 2.75 in the t-test (**Table 2**).

**Table 1:** List of ten SNPs with LOD (-log(P-value)) greater than 5.83 detected by one or more models (FarmCPU or GLM) in GAPIT 3, along with t-test results for salt tolerance.

SNP	Chr	Pos	MAF %	LOD [-log(P-value)]	Model	Lod_ (*t*-test)	Beneficial _allele	unbeneficial _allele	Linked gene (0-4kb)
Gm01_4306329_ss715579436	1	4306329.00	22.29	6.82	GLM	7.26	A	G	Glyma.01G039600 Glyma.01G039700
Gm01_4312808_ss715579441	1	4312808.00	23.89	6.81	GLM	7.30	A	G	Glyma.01G039700 Glyma.01G039800
Gm01_4336306_ss715579451	1	4336306.00	22.38	6.60	GLM	6.58	G	A	Glyma.01G040000
Gm02_36878905_ss715582154	2	36878905.00	48.49	7.45	GLM	4.11	G	A	Glyma.02G195400
Gm02_36940321_ss715582156	2	36940321.00	34.99	6.73	GLM	4.42	A	G	
Gm02_36991983_ss715582157	2	36991983.00	34.81	6.87	GLM	4.52	A	G	
Gm03_43213208_ss715586397	3	43213208.00	45.74	5.85	GLM	8.97	T	G	Glyma.03G230400 Glyma.03G230500 Glyma.03G230600
Gm03_43220331_ss715586399	3	43220331.00	44.85	6.11	GLM	9.33	C	T	Glyma.03G230600 Glyma.03G230700
				6.03	FarmCPU	9.33			
Gm07_41776639_ss715598058	7	41776639.00	43.78	6.44	GLM	5.94	T	C	
Gm16_7722217_ss715625494	16	7722217.00	11.55	5.95	FarmCPU	2.75	T	C	Glyma.16G076200 Glyma.16G076300

Each SNP is located on a chromosome, and its position is based on the soybean genome reference Wm82.a2 and its linked genes within less than 4 kb. Chr, chromosome; Pos, position; MAF, minor allele frequency; LOD, Logarithm of the Odds.

**Table 2:** Genomic prediction (r-value) of salt tolerance using nine SNP sets: eight randomly selected SNP sets ranging from 10 to 10,000 SNPs (r10 to r10000), plus the GWAS-derived SNP marker sets (10 markers - m10).

GP Model	r-value						SE of r-value					
	rrBLUP	BA	BB	BL	BRR	SNP.set Mean	rrBLUP	BA	BB	BL	BRR	SNP.set Mean
**r10**	0.20	0.20	0.14	0.20	0.19	0.19	0.067	0.072	0.088	0.079	0.083	0.078
**r100**	0.17	0.24	0.22	0.24	0.22	0.22	0.083	0.076	0.075	0.084	0.070	0.078
**r200**	0.24	0.24	0.25	0.26	0.24	0.25	0.070	0.087	0.086	0.078	0.081	0.080
**r500**	0.28	0.29	0.29	0.30	0.29	0.29	0.081	0.090	0.084	0.071	0.092	0.084
**r1000**	0.30	0.31	0.29	0.30	0.31	0.30	0.074	0.077	0.067	0.076	0.075	0.074
**r2000**	0.33	0.30	0.29	0.31	0.31	0.31	0.085	0.087	0.087	0.077	0.076	0.083
**r5000**	0.32	0.28	0.30	0.29	0.31	0.30	0.072	0.077	0.085	0.081	0.078	0.079
**r10000**	0.30	0.27	0.28	0.27	0.30	0.28	0.083	0.084	0.072	0.075	0.080	0.079
**m10**	0.36	0.38	0.38	0.40	0.39	0.38	0.082	0.079	0.088	0.073	0.077	0.080
**GP.model** **mean**	0.28	0.28	0.27	0.29	0.28	0.28	0.077	0.081	0.081	0.077	0.079	0.079

Predictions were estimated using five genomic prediction (GP) models: rrBLUP, BA, BB, BL, and BRR. The standardized errors of the r-values (SE) are also listed. r-value, the genomic prediction r value for salt tolerance; SE of r-value, the standard error of the genomic prediction r value for salt tolerance; rrBLUP, ridge regression best linear unbiased prediction; BA, Bayes A; BB, Bayes B; BL, Bayes LASSO; BRR, Bayes ridge regression; SNP.set Mean, the average values of the five different genomic prediction models; r10 - r10000, the SNP sets randomly selected in quantities ranging from 10 to 10,000.

### Candidate genes for salt tolerance

Eleven genes are located within 5 kb upstream and downstream of 7 of the 10 SNP markers associated with salt tolerance (**Table 1**; **Supplementary Table 2**). Gene information is based on the soybean reference genome *Glycine max* Wm82.a2.v1 (https://phytozome-next.jgi.doe.gov/info/Gmax_Wm82_a2_v1).

The four genes, *Glyma.01G039600* (leucine-rich repeat receptor-like protein kinase family protein), *Glyma.01G039700* (Vps51/Vps67 family protein, components of vesicular transport), *Glyma.01G039800* (galactosyltransferase family protein), and *Glyma.01G040000* (glutathione S-transferase TAU 18), are physically close to the three SNP markers—Gm01_4306329_ss715579436, Gm01_4312808_ss715579441, and Gm01_4336306_ss715579451— (**Tables 1**, **2**).

Similarly, candidate genes *Glyma.02G195400* (syntaxin of plants 121), located at 36,872,648 bp to 36,875,320 bp on chromosome 2, *Glyma.03G230400* (invertase H), *Glyma.03G230500* (plus-3 domain-containing protein), *Glyma.03G230600* (protein of unknown function, DUF538), and *Glyma.03G230700* (importin alpha isoform 4) on chromosome 3 and *Glyma.16G076200* (pyrimidin 4) and *Glyma.16G076300* (long-chain fatty alcohol dehydrogenase family protein on chromosome 16 were within physically close to the significant SNPs on those chromosomes (**Tables 1**, **2**).

### Genomic prediction

*GP in the reference:* The GP analysis yielded moderate to high r-values of 0.46, 0.60, and 0.44 for the maBLUP, gBLUP, and sBLUP models, respectively. These estimates were obtained using a training set to predict salt tolerance in a panel of 563 soybean germplasms genotyped with 34,181 SNPs in (**Supplementary Figure 5**). These results indicate that GS is effective for salt tolerance selection.

**Figure 4:**
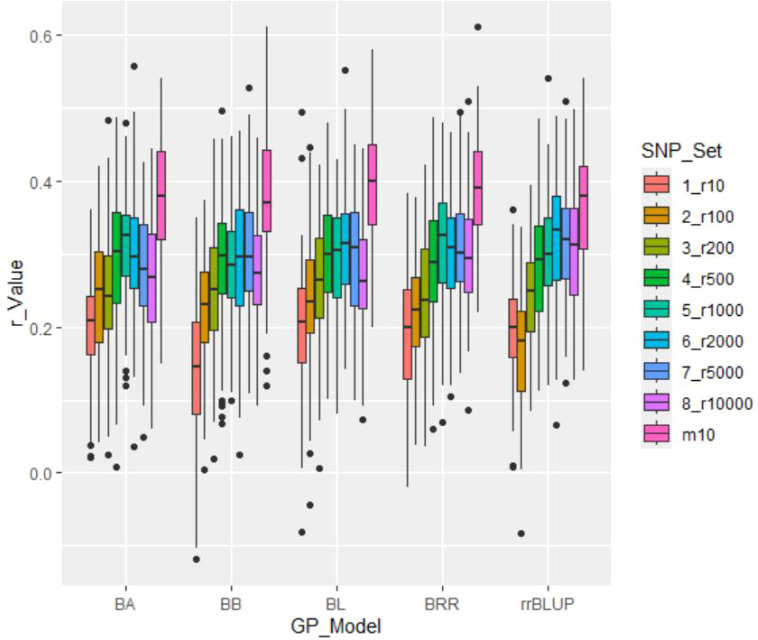
Genomic prediction (r-value) of salt tolerance using nine SNP sets: eight randomly selected SNP sets ranging from 10 (r10) to 10,000 SNPs (r10000), plus the GWAS-derived SNP marker sets (10 markers - m10). Predictions were estimated using five genomic prediction (GP) models: BA, BB, BL, BRR, and rrBLUP.

*GP in cross-prediction using randomly selected SNP markers*: GP using randomly selected SNP markers for cross-prediction yielded the following average r-values: 0.19 (ranging from 0.14 to 0.20) for the 10-SNP set (r10); 0.22 (ranging from 0.17 to 0.24) for the 100-SNP set (r100); 0.25 (ranging from 0.24 to 0.26) for the 200-SNP set (r200); 0.29 (ranging from 0.28 to 0.30) for the 500-SNP set (r500); 0.30 (ranging from 0.29 to 0.31) for the 1,000-SNP set (r1000); 0.31 (ranging from 0.29 to 0.33) for the 2,000-SNP set (r2000); 0.30 (ranging from 0.28 to 0.32) for the 5,000-SNP set (r5000); and 0.28 (ranging from 0.27 to 0.30) for the 10,000-SNP set (r10000) (**Table 2**; **Figure 4**). These results demonstrate that the r-value increased with the number of randomly selected SNPs, with an average r-value rising from 0.19 in the 10-SNP set to 0.30 in the 10,000-SNP set. This suggests that a randomly selected SNP set consisting of at least 1,000 SNPs (r = 0.30) should be used in GS for selecting salt tolerance.

*GWAS-derived SNP marker set*: The average r-value was 0.38, ranging from 0.36 to 0.40, for the 10-marker set (m10) (**Table 2**; **Figure 4**). These results indicate that the r-value was moderately high, exceeding 0.35 and surpassing those of SNP sets randomly selected from 10 SNPs to 10,000 SNPs. This suggests that GWAS-derived SNP markers can be effectively used for GP and for selecting salt tolerance in soybean breeding through MAS and GS.

*GP Model*: All five GP models—BA, BB, BL, BRR, and rrBLUP—exhibited similar r-values, indicating that each model is effective for selecting salt tolerance in GS.

### Genetic diversity and utilization of the salt tolerant germplasms

The phylogenetic analysis showed that the 150 salt-tolerant germplasms were distributed throughout the tree of 563 germplasms and did not separate into distinct groups of susceptible and tolerant germplasms (**Supplementary Figure 6**). This indicates that the 150 salt-tolerant germplasms have broad genetic backgrounds. Further analysis revealed that these 150 salt-tolerant germplasms can be divided into three distinct groups (**Supplementary Figures 7**, **8**), confirming that they possess different genetic backgrounds.

**Figure 5:**
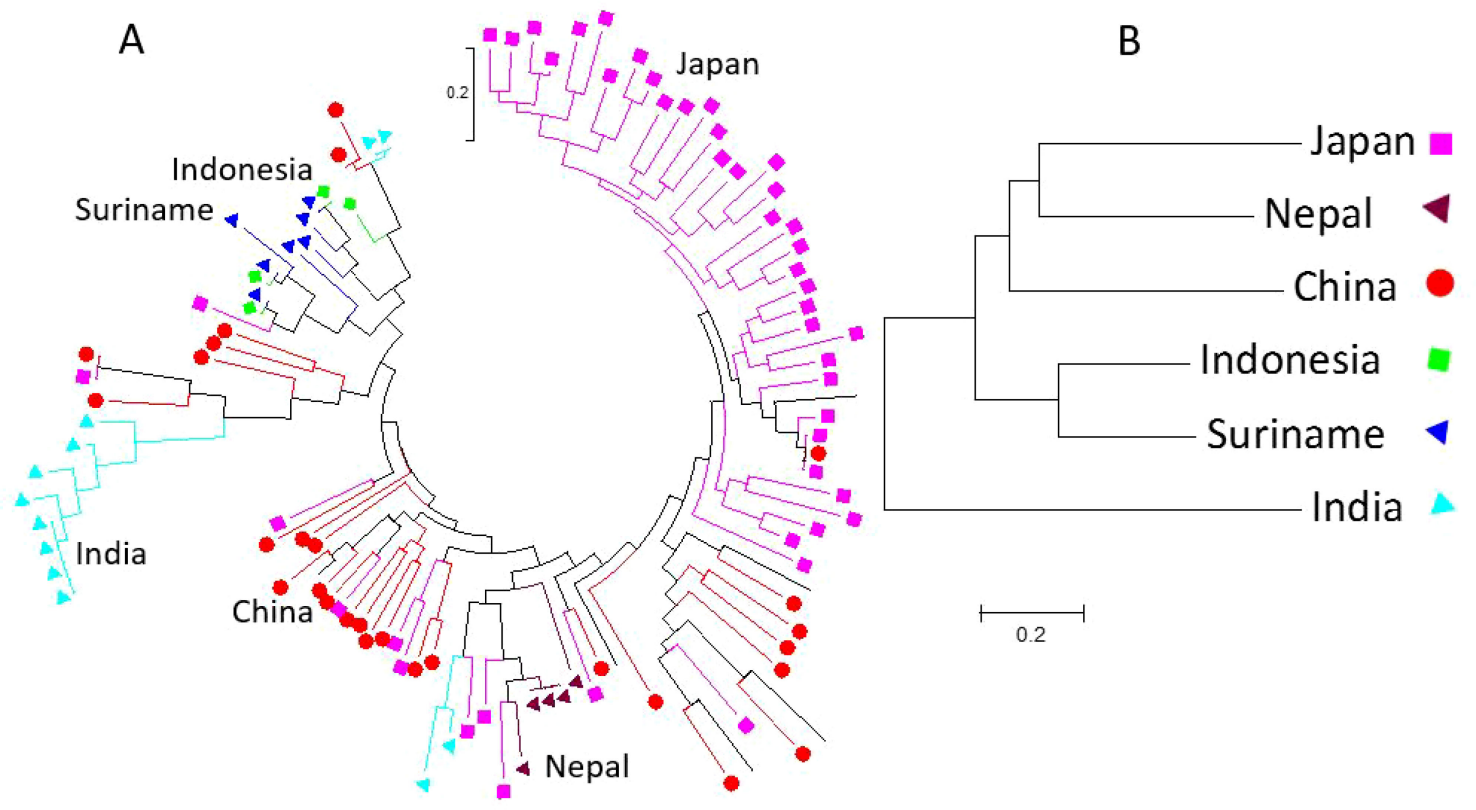
The non-taxon ring phylogenetic tree of 104 salt-tolerant soybean germplasms was constructed using the Maximum Likelihood (ML) method in MEGA 7, based on 6000 randomly selected SNPs distributed across 20 soybean genome chromosomes **(A)**. The colored shapes and branches represent germplasms from one of the six countries: Japan, Nepal, China, India, Suriname, and Indonesia. The traditional phylogenetic tree of soybean germplasm from the six countries is shown in **(B)**.

Among the 150 salt-tolerant germplasms, six countries contributed a total of 104 germplasms: Japan (46), China (31), India (12), Suriname (7), Indonesia (6), and Nepal (5) (**Supplementary Table 1C**). Phylogenetic analysis of these 104 germplasms showed that germplasms from the same country generally clustered together (**Supplementary Figure 9**), suggesting that germplasms from the same country share similarities in genetic backgrounds. Specifically, germplasms from Nepal are closer to those from Japan, followed by China; germplasms from Indonesia are closer to those from Suriname; while germplasms from India are more distinct (**Figure 5**). This clustering suggests that geographic factors influence the distribution of salt-tolerant germplasms.

## Discussion

Soybeans are a crucial source of plant protein, accounting for over 60% of daily plant protein consumption (Sedivy et al., 2017). With the increasing global demand for food, soybean production must be enhanced to meet the rising need for plant protein (Lu et al., 2021). However, soybean yields are highly susceptible to adverse environmental conditions, with salt stress being a significant abiotic factor that severely impacts soybean production and poses a substantial threat to agricultural productivity (Leng et al., 2021; van Zelm et al., 2020). Identifying genes associated with salt stress tolerance is essential for developing salt-tolerant soybean varieties and improving soybean yields. Although some genes regulating salt tolerance traits have been reported, research on soybean salt tolerance remains insufficiently comprehensive. Therefore, this study aims to analyze the salt tolerance of 563 soybean germplasms resource from the USDA GRIN, identify salt tolerance-related genes, and conduct genomic predictions. These efforts are vital for selecting salt-tolerant soybean germplasm and breeding salt-tolerant soybean varieties.

In recent years, numerous QTLs associated with soybean salt tolerance have been identified. However, due to the complex nature of soybean salt tolerance, which is controlled by multiple genes, the related loci and candidate genes identified vary across different populations or using different analytical methods. This study conducted a GWAS on 34,181 SNP markers, identifying 10 SNPs associated with salt tolerance, located on chromosome 1, 2, 3, 7, and 16. In both GAPIT models (GLM and FarmCPU), a locus on chromosome 3 showed LOD scores exceeding 5.83 and t-test values greater than 8.9, indicating a robust QTL for salt tolerance. Four genes—*Glyma.03G230400*, *Glyma.03G230500*, *Glyma.03G230600*, and *Glyma.03G230700*—are closely linked to SNP markers Gm03_43213208_ss715586397 and Gm03_43220331_ss715586399 within 4 kb. Previous studies have also identified salt-tolerant genes in this region (Kan et al., 2015; Patil et al., 2016; Zeng et al., 2017). On chromosome 2, three SNP markers—Gm02_36878905_ss715582154, Gm02_36940321_ss715582156, and Gm02_36991983_ss715582157—within the 36,878,905 bp to 36,991,983 bp region exhibited LOD scores greater than 6.7 and t-test values exceeding 4.1, suggesting a salt tolerance QTL. The gene *Glyma.02G195400* is closely linked to SNP marker Gm02_36878905_ss715582154, within 4 kb. Similar QTLs have been reported by Zeng et al. (2017) and Kan et al. (2015). On chromosome 1, three SNP markers—Gm01_4306329_ss715579436, Gm01_4312808_ss715579441, and Gm01_4336306_ss715579451—located within the 4306329 bp to 4336306 bp region showed LOD scores greater than 6.5 and significant t-test values, indicating a salt tolerance QTL. Four genes—*Glyma.01G039600*, *Glyma.01G039700*, *Glyma.01G039800*, and *Glyma.01G040000*—are closely linked to these markers within 5 kb. Similar QTLs have been identified in rice by Pandit et al. (2010). On chromosome 7, SNP marker Gm07_41776639_ss715598058 at 41,776,639 bp showed an LOD score of 6.44 and a t-test value of 5.94, indicating significant association with salt tolerance. This region was also identified by Zeng et al. (2017). The SNP marker Gm16_7722217_ss715625494 on chromosome 16, located at 7,722,217 bp, exhibited an LOD score of 5.95 and a t-test value of 2.75, suggesting a salt tolerance QTL. Two genes—*Glyma.16G076200* and *Glyma.16G076300*—are closely linked to this marker within 3 kb. Similar QTLs in this region were identified by Zeng et al. (2017).

In this study, the accuracy of GP was evaluated by assessing the correlation coefficient (r) between the GEBV and the observed values. (Bhattarai et al., 2022; Joshi et al., 2022) Initially, salt tolerance of 563 soybean germplasms were predicted using three different genomic prediction models: maBLUP, gBLUP, and sBLUP for itself by cross-population prediction. The r values obtained from these models were 0.46, 0.60, and 0.44, respectively, indicating that genomic selection for salt tolerance is effective. Subsequently, cross-prediction was conducted using randomly selected SNP markers and GWAS-derived SNP marker sets. The results showed that the r-values were relatively higher for the GWAS-derived SNP marker sets. All five GP models—BA, BB, BL, BRR, and rrBLUP—exhibited similar r values, demonstrating that each model is effective for selecting salt tolerance through GS. These findings suggested that GP and salt tolerance selection can be effectively utilized in soybean breeding through MAS and GS.

## Conclusion

Through GWAS analysis of 150 tolerant and 413 susceptible germplasms with 34,181 SNP loci, we identified 10 SNPs associated with salt tolerance: four SNP markers on chromosome 1 (Gm01_4306329_ss715579436, Gm01_4312808_ss715579441, and Gm01_4336306_ss715579451), three markers on chromosome 2 (Gm02_36878905_ss715582154, Gm02_36940321_ss715582156, and Gm02_36991983_ss715582157), two markers on chromosome 3 (Gm03_43213208_ss715586397 and Gm03_43220331_ss715586399), and one marker each on chromosomes 7 and 16 (Gm07_41776639_ss715598058 and Gm16_7722217_ss715625494, respectively). We assessed the accuracy of GP by examining the correlation coefficients (r) between GEBV and observed values. Using different GP models and SNP sets, we observed that r-values were up to 0.4 when using significant SNP markers derived from GWAS. The information provided valuable references for selecting and breeding soybean varieties with enhanced salt tolerance.

## Data Availability

The original contributions presented in the study are included in the article/**Supplementary Material**. Further inquiries can be directed to the corresponding authors.
